# PD86 Saúde e Educação In Access To Knowledge About Health Technology Assessment In Brazil

**DOI:** 10.1017/S0266462325102134

**Published:** 2025-12-29

**Authors:** Jéssica Evelyn de Andrade, Mariana Dias Lula, Juliana Álvares-Teodoro, Augusto Afonso Guerra-Junior, Cristina Mariano Ruas

## Abstract

**Introduction:**

Saúde e Educação (SeE) is an extension project of the Federal University of Minas Gerais (UFMG), Brazil, which aims to disseminate information and research related to health in a simplified way for the public. Based on the perception of existing knowledge gaps in society, the SeE created a thematic series to disseminate accessible information about health technology assessment (HTA).

**Methods:**

A partnership was established between the Collaborating Centre for Health Technology Assessment and Excellence (CCATES) and the UFMG. Members of CCATES were invited by the SeE to voluntarily produce informative texts about HTA to be published and disseminated by the SeE. The SeE editors reviewed the texts and formatted them to have a layout that motivates reading and facilitates understanding. The materials were published on the SeE website and disseminated on the project’s social networks. Strategic days were selected for publication. Partnerships with patient associations, for example, were formed to facilitate dissemination. The project had no funding.

**Results:**

In 2024, five texts by seven collaborators were produced, published, and disseminated. Three are in the process of preparation or review. To date, the texts have received 154 views on the website, and the advertisements have received more than 1,622 views on social media. Challenges encountered include problems with knowledge translation and reaching the target audience.

**Conclusions:**

The SeE has been disseminating knowledge about HTA through digital means. This is important to ensure that information is accessible to the public. It can promote more inclusive and participatory decision-making, contribute to equity in access to health innovations, strengthen social control, and improve the implementation of technologies that meet the population’s needs.

